# Hospital and Institutional News

**Published:** 1915-09-25

**Authors:** 


### HOSPITAL AND INSTITUTIONAL NEWS.

### THE ROYAL VISIT TO EXETER.

DURING their recent tour in the West the King and Queen visited two of the five Voluntary Aid hospitals at Exeter and expressed themselves as exceedingly pleased with the appearance and equipment of the hospitals and everything they had seen, and the Queen, in taking leave of the Mayoress, spoke of her depôt for the provision of comforts and hospitality to soldiers. Her Majesty was evidently conversant with the details of the work and the devotion of the ladies of Exeter. The total number of beds in the Exeter hospitals is 570, of which 520 are at present occupied. This group of hospitals form together a central hospital to which twenty other Voluntary Aid hospitals and sixteen convalescent homes in the county are affiliated. The grand total of beds is 1,364, of which 1,085 are at present occupied. The total number of patients admitted to date is: In Exeter, 3,343; outside Exeter, 1,894; total, 5,237. The King expressed warm appreciation of the work that is being done.

### THE ZEPPELIN AND THE CHILDREN.

IT is recorded that in one hospital situated in a district recently visited by hostile air-craft, where the children were becoming alarmed, the night sister suggested that it was an extra "Guy Fawkes" night. She then produced some sweets, the little ones were persuaded to listen to the "fireworks," and so happily were soon asleep.

### POISON IN AEROPLANE CONSTRUCTION.

UP to the present during this year nineteen non-fatal and four fatal cases of poisoning by dope containing tetrachlor-ethane used in aeroplane construction have been notified to the Home Office. According to a statement made in the House of Commons by Mr. Brace on Thursday last week, adequate precautions are being enforced by the Factory Department in all works in which the process of doping is carried on, so that we may expect to see a diminution of the number of poisoning cases due to this cause. If it is found that in spite of these precautions accidents still occur, further encouragement will be given to the research that is now going on with the object of finding a substitute for this dangerous dope. It is said that certain firms are already employing a dope that does not contain tetrachlor-ethane, and if aeroplanes so treated are found satisfactory, so much the better.

### THE END OF CLEVELAND STREET INFIRMARY.

THE Holborn Board of Guardians have decided to close their infirmary at Cleveland Street, London, W., on the 30th inst. This institution, which was formerly known as the Central London Sick Asylum, under the St. Giles' Board of Guardians, came under the administration of Holborn at the amalgamation of these parishes. The Guardians propose to transfer the officers from Cleveland Street (as far as is practicable) to their other institutions. Permanent officers for whom a transfer is not available will probably receive a compensation allowance, whilst temporary officers will, of course, leave at the termination of their engagement on the 30th inst. It has not yet been decided to what purpose the building will be put, but we understand that the Guardians will seek the opinion of the Local Government Board on the matter.

### GERMAN AUTHORITIES UNEASY!

THE medical profession in Germany, or rather that portion of it which is still engaged in civil practice, has apparently become less keen than usual on pathological specimens, and the number of throat swabs, blood tests, fluids for cytological diagnosis, and suchlike, sent to laboratories, has fallen very considerably of late. This fact has caused much uneasiness to the authorities, who regard such measures as of great importance in controlling epidemics of infectious disease and in securing early diagnosis of serious illness. In this they are no doubt perfectly right. It is interesting to note, however, that it has been left to the Prefect of Police to notify the profession in Berlin, and to declare that however much the ranks of the medical men may be depleted by the demands of the army, the authorities cannot allow practitioners to neglect such important procedures.

### D.S.O. FOR A NAVAL SURGEON.

A SPECIAL supplement of the *London Gazette* recently issued contained a long list of naval awards for gallantry and good service. Among them we are glad to see the name of Surgeon Basil Alfred Playne, R.N., R.N.D., who receives the Distinguished Service Order for gallantry and good service during operations near Gaba Tepe from April 28 to May 1. On several occasions, it is recorded, he rushed across the open (the communication trench being incomplete) into the fire trenches, and attended the seriously wounded, regardless of the severity of the enemy's fire. On one occasion he carried a wounded officer on his back from the fire trench to the communication trench under heavy fire, and his conspicuous bravery not only inspired the stretcher-bearers to perform fine work, but gave confidence and spirit to all ranks. He was again several times brought to notice for gallant deeds when attending wounded on May 3 and 4.

### ECONOMY IN POOR-LAW DENTISTRY.

THE wave of economy that has swept over the country has affected the Southwark Board of Guardians. A patient sent by them to the Lancing Home of Rest, Sussex, has to have four teeth extracted, and the Guardians have been notified that the charge made by the dentist at Lancing will be 2s. 6d. for each extraction. The Guardians have come to the conclusion that this charge is excessive, especially when they can have teeth drawn at a shilling each in South London. They have decided to ascertain from the medical attendant at the Home whether it is essential that the teeth should be extracted, and, if so, the patient is to be sent to their infirmary at London for the operation to be performed. By an arrangement with the railway authorities the patient will be able to make the return journey for 5s., so that with the additional 4s. to be paid for the extractions in London, there will be a saving to the ratepayers of a shilling. To effect this the patient will have to travel over one hundred miles in his journey to and from Lancing. Where does the patient come in?

### A BROTHERLY ACTION.

Freemasons, individually and collectively, who have done much in these days of stress to alleviate suffering, are anxious to do more. In accordance with this benevolent design the London Rank Association of Past Masters has inaugurated a visiting organisation for the benefit of Freemasons and the sons of Freemasons temporarily located in London military hospitals, and any Masonic sailor or soldier, or son of a Freemason, at any of the London military hospitals may, if he intimates a request to that effect, receive a visit from one of the London brethren. It should be understood that unless a personal request for a visit be made to the Secretary of the London Rank Association, Connaught Rooms, Great Queen Street, W.C., and a pass for admission be sent, the visiting Master cannot make promiscuous application and interrupt the hospital authorities in their work. This desire of Freemasons to render every kind of assistance in their power is in keeping with the traditions of the craft.

### WAR BONUSES FOR NURSES.

Lambeth Guardians have decided to solve the problem of the dearth of nurses by the granting of war bonuses. This was suggested by Dr. Baly, the medical superintendent, who in a report stated: "The shortage of trained nurses at the infirmary is becoming serious. At the present time there are only twenty instead of the allowance of thirty, and there is a prospect of a further reduction. In the circumstances, it has been necessary to increase the responsibilities of those remaining to a considerable degree, and I recommend that a war bonus should be granted them. I am further of opinion that the fairest method would be to give a six-monthly bonus equivalent to 33⅓ per cent. of the salary received by each trained nurse during that period. On account of the reduction in numbers, the total cost of the nursing staff will not exceed the amount allowed in normal times, and the staff nurses will receive a salary which will not exceed that which can be obtained in a military hospital." The officers who will participate in the war bonus are the head-nurses, whose present salaries are at the rate of £35 a year, rising by yearly increments of £2 to a maximum of £40; and staff nurses, whose salaries are £30, rising to £35.

### HOMES OF REST—A HAPPY EXAMPLE.

A notable addition has just been made to the institutions of Hull by the opening of the Lee's Homes of Rest. This institution is situated amid ideal surroundings, with a restful aspect that will be appreciated by the old people who spend their declining years within the precincts of the new buildings. The institution is named after the late Dr. Lee, who left a legacy of £200,000 for the purpose of building and endowing it. During the last few years of his life this great benefactor, who lived to the ripe old age of eighty-six, was obsessed with the wish to benefit a class not hitherto provided for—namely, those who had been reduced from better circumstances. The scheme he devised was for providing Rest Homes to accommodate a hundred persons—fifty single and twenty-five married couples—with power to extend if the income [illegible] sufficient. Fortunately for the people whom [illegible] Lee desired to benefit, the income was found sufficient for a much larger scheme, and the buildings have been erected to accommodate eighty single persons and forty married couples, who will be able to spend the evening of their lives under favourable conditions. We hope that when the great war is over, Dr. Lee's example may find many imitators, or possibly State support in the new England yet to come.

### "VOLUNTARY CONSCRIPTION" (?)

At a meeting of the War Emergency Committee, held recently at the offices of the British Medical Association, some most definite statements were made as to the needs of the Army for further supplies of medical officers, and an interesting plan was propounded for meeting them. It was stated that by the middle of January at least 2,000 additional doctors will be required, and that at the present rate of entry not half that number is likely to be recruited. The Committee has decided to initiate an effort which the *Lancet* characterises as "voluntary conscription." The essence of this scheme is that each division of the British Medical Association is informed how many doctors its area is expected to furnish, and local committees are to use all the arts of peaceful persuasion to enrol the requisite number. In particular, great efforts are to be made to ensure that colleagues in the neighbourhood of each doctor's practice shall preserve his connection for the absentee as far as possible. It cannot be said that the voluntary system is being given a fair chance in the medical profession; indeed, it is difficult to avoid the belief that this plan will amount in many instances to nothing less than veiled compulsion.

### CHANGES IN DIETARY AT INFIRMARIES.

Apropos of the need for economy by Poor-Law Guardians to which reference has recently been made in The Hospital, it is interesting to note that various boards are endeavouring to meet the changed conditions of the times by making substitutes in the dietary scales for those in their institutions. Thus the Southwark Board of Guardians have decided to provide one day a week Australian rabbits in place of the usual meat for dinner, whilst the Wandsworth Board have substituted margarine for butter for their inmates, but have declined to sanction a proposal that the officers should also have margarine. This suggests the observation that what is good enough for the patients in the [illegible]ary should also be good enough for healthy [illegible]le. It is common knowledge that many people of [illegible]e professional classes are substituting margarine for butter in their own households, and there is only the question of sentiment that prevents the more general use of margarine at this time. The Metropolitan Asylums Board have also made a number of changes, which not only tend to economy, [illegible] provide a more varied diet for everyone concerned. In the infirmaries the medical superintendents have full power to order what they think is absolutely necessary for their patients, and con[illegible]ently there is little fear of the patients suffer[illegible] the proposals of those in authority.

### NOTABLE ABSENCE OF APPREHENSION.

A vacancy has occurred for an assistant tuberculosis officer to the Lambeth Municipal Tuberculosis Dispensary, Dr. S. Nicol Galbraith, the present occupant of the office, having been ap[illegible]nted medical officer of health at Newark-on-[illegible]. The Public Health Committee recom[illegible]d a salary of £400, rising to £500 by [illegible] increments of £25. The General Purposes [illegible]ttee of the Borough of Lambeth, however, [illegible] the emoluments to £300, rising to £400, a [illegible] which was upheld by the Borough Council despi[illegible]e protest of Dr. McKeith. He pointed out t[illegible]he usual rate of remuneration for these appointments was £500 per annum, and that there was no possibility of obtaining candidates at £300 a year. The majority held that a woman doctor would be procurable at the lower remuneration, and the Council coincided in this view. London has good reasons to be dissatisfied with the action and work of the Borough Councils within its area, and this marked instance of lack of apprehension of the market value of medical services and of medical rates of remuneration with the dearth of candidates is as remarkable as it is lamentable. We are confident that women graduates and their leaders will have pleasure in demonstrating to the Lambeth Council the hopeless ignorance they have exhibited in this matter.

### THE IMPORTANCE OF ORAL HYGIENE.

It is disappointing to find that the importance of oral hygiene is but slowly impressing itself upon the minds of tuberculosis officers. Perhaps it were fairer to credit them with realising how advantageous it is for consumptives to possess adequate and healthy teeth, but then it must be admitted that as medical officers they have not succeeded in impressing the dental point on their committees. A tuberculosis officer cannot be expected to do anything for a patient's mouth except the most urgent extractions and the antiseptic treatment of septic gums. It is usually stated on admission forms for sanatoria that the teeth must be put in good order before the patient enters for treatment, and this is implied in the case of London insured tuberculous persons. But for the lack of opportunity or time it happens that many a patient is sent off to undergo sanatorium treatment with a high degree of gingival inflammation or teeth that are a hindrance and not a help to mastication. Unless the medical officers of the institution are keen on minor dental surgery, these unfortunate persons are told to get fat, and their dyspepsias are attacked with "Mist. Gent. Alk." Those who are fostering antituberculosis measures will find the expenditure of a comparatively small sum in securing the services of a dental surgeon to be a profitable investment as measured by the gain in health to the patients recommended for sanatorium benefit. Not a few so-called early cases of tubercle are suffering from unhealthy mouths and nothing else.

### POOR-LAW GUARDIANS AND ECONOMY.

As a general rule there have been considerable reductions in the amounts estimated to meet the requirements of Metropolitan Boards of Guardians, which will mean a slight reduction in the local rates. The diminution in expenditure is in a great measure due to the reduced number of persons dependent on the Poor Law, though this has to a considerable degree been minimised by the extra cost of provisions, drugs, and other necessaries. The Lambeth Board of Guardians have reduced their precept £6,000 as compared with the same period last year, and Camberwell Board of Guardians show a decrease of £6,500, whilst the Southwark Board of Guardians will be content with the same amount they had last year. Whilst there are these reductions on nearly every hand, it is all the more surprising to find that the Bermondsey Board of Guardians are asking their Borough Council for £82,000 to meet their liabilities in the next six months. This is an increase of £12,500 on the sum required last year, the total amount for the whole year being £164,000, or £25,000 more than in 1913-14. The Board have no building schemes in contemplation, and there has been a decrease in the number of recipients of their assistance, so that the great increase in the sum required is almost unexplainable when the diminished demands of neighbouring parishes are taken into account. It is to be hoped that the huge amount will not be required, but that is no excuse for asking for it when economy should be the keynote of all public work at the present time. Will the large demand of the Bermondsey Board be sanctioned?

### THE WORK OF THE FRENCH RED CROSS.

Some interesting particulars are to hand concerning the work of the French Society for aiding wounded soldiers. During the ten months which elapsed between the opening of hostilities and June 1, 1915, the "Société Française de secours aux blessés militaires," which is one of the three societies composing the French Red Cross, had organised 773 auxiliary hospitals, representing 70,000 beds, ninety-three first-aid posts, eighty-eight infirmaries, and forty-five railway station canteens where the sick and wounded receive the necessary care and soldiers passing through are fed. In these hospitals are employed 12,000 trained and qualified nurses, and 10,000 assistants give their aid in the kitchens, linen rooms, and sterilising rooms. Sixteen of these nurses and assistants had paid for their devotion with their lives by June 1; five having been killed during the bombardment of Rheims, and the others having succumbed to contagious diseases contracted while nursing the wounded. Among the nurses of the Society one has received the medal of the Legion of Honour, thirty have been mentioned in the orders of the day, and thirty-three have received other medals. The Society has organised a rapid transportation service for the wounded, which consists of eighty-eight automobiles divided into a certain number of units. It has also sent many articles of clothing and comforts for the sick to the hospitals and the ambulances and to the Front. In order to perform all these services the Society has expended the sum of over £800,000 sterling. At the beginning of the war the Society had at its disposal about £3,000; the rest it owes to the generosity of the public. Truly a wonderful record!

### MOBILE LABORATORIES AT THE FRONT.

The special correspondent of the *Morning Post*, in an article dated from the British Headquarters in France, pays a warm compliment to the value and efficiency of the mobile laboratories which have gradually been evolved in the circumstances produced by trench warfare. The first attempt was in the shape of a motor-bicycle and a side-car, but gradually the development has now attained the proportions of an "ample lorry." The correspondent adds that the search for "carriers" of the germ of cerebro-spinal meningitis proved so successful that it had to be abandoned. In other words, the "carriers" proved to be so abundant that any attempt to isolate them was out of the question. A febrile condition fairly common in the trenches, and resembling an attack of influenza, is called by the soldiers, with a sort of ghastly humour, "liquid German fever," on the ground that it is due to the odour from putrefying corpses. The work of the bacteriological laboratories evidently makes a strong appeal to the correspondents of the lay papers. A very graphic description of the "germ seekers" has appeared in the *Daily Telegraph*, where we are introduced, amongst others, to "O. C. Mosquitos," who has come from Singapore, and who, though he does not find his usual prey, takes steps to get rid of the stagnant pools and filthy lairs in which the mosquito is prone to breed. Tetanus, it is said, is scotched by the serum treatment, but the disappearance of cerebro-spinal meningitis is attributed to the weather. "The young scientists," the correspondent writes, "do not stand to gain much, but they go on working quietly with their microscopes and test tubes among all the muck and blood and agony of war."

### INFANT WELFARE.

In Liverpool the subject of infant welfare has always been regarded as of vital importance, and the Notification of Births Act has been in force in that city for several years, so that no extensive changes are called for now that the Act has been made compulsory. Dr. E. W. Hope, the medical officer of health, has recently declared that the care of junior citizens should be looked upon as the main-spring of all the work of the public health department. Housing and town planning operations, and even the cleansing of the streets, have a bearing, and a very direct one, on this activity. In Liverpool five infant welfare centres are in existence, besides several schools for mothers. For the benefit of the "ex-baby" day nurseries are provided where he can wriggle and cry in companionable society; the "ex-baby" sometimes has a rather poor time, as he is too young to take care of himself and the newcomer's demands mean that he has to take a back seat. The value, therefore, of day nurseries needs no emphasising, and they should be included in any scheme for the promotion of infant welfare. In Liverpool efficient midwifery is one of the chief concerns of the child welfare scheme. Most of the 208 midwives are fully trained, and are banded together in a sort of trade union society which is encouraged by the authorities. This association forms a medium of communication with the sanitary officials, and through it the women are enabled to improve and keep up their professional knowledge by means of lectures and discussions. In Liverpool it appears that midwives play a more considerable part in the infant welfare scheme than in most centres: the policy is one which is yielding excellent results among the rising generation.

### YORK COUNTY HOSPITAL.

In our issue of the 11th instant, page 495, we drew attention to a published statement that the York County Hospital has been labouring for years under a debt of £3,900. At the quarterly court of the governors on the 14th inst. lengthy speeches of congratulation were made owing to the announcement that £1,701 had been promised, including £500 from Mr. Rutson. How much of this £1,701 was given towards the debt and how much towards current expenses was not stated. Mr. Melrose referred to "the very generous public," whilst he stated the hospital was still in debt, and, "so far as his experience went, was always likely to be so." Mr. Melrose's words demonstrate the absence of a sound financial policy. That policy's absence is due to the want of business aptitude in those responsible for the administration of the county hospital, which is situated in one of the most prosperous cities in the North of England. We commend to the independent governors and people of York the words of Mr. C. W. Bowerman, M.P., in his advice to the Cabinet: "Stop this talking, and for Heaven's sake get on with the business management of the [hospital]. For years we have heard nothing but talk, talk, talk. What a laughing-stock we must be truly!" Proceedings in connection with the York County Hospital, its chronic impecuniosity, and relative inefficiency have made it the laughing-stock of all who know what the financial strength of a county hospital, properly administered, can be and ought to be. It is noteworthy in this connection that not a word of explanation was given at the court on the 14th inst. as to how the committee have managed to place 120 out of 155 available beds at the disposal of the War Office without interfering with the claims of the civilian population.

### SOLDIERS WHO REFUSE TREATMENT.

The French Minister of War has on various occasions been consulted with regard to soldiers who refuse immunising treatment against certain diseases, or operations for the amelioration of conditions produced by wounds, and according to the Paris correspondent of the official organ of the American Medical Association, he has sent certain instructions on the subject to army doctors. In the first place he very wisely advises that in general it is important to avoid any coercive measures. But in a case of obstinate refusal after persuasive methods have been tried, he points out, in the official instruction, that the solution of the problem varies according to the factors entering into it. When it is a question of legally prescribed measures involving both individual and collective prophylaxis, such as vaccination against smallpox or typhoid, it is not permissible to decline the treatment. In the interests of the community, the treatment should be applied without exception, and refusal to submit may be treated as a breach of military discipline. When a wounded soldier refuses a simple and bloodless method of treatment which tends to ameliorate the patient's infirmities without any risk whatever, and to reduce his incapacity for labour, he may be treated as a malingerer who intentionally prolongs or aggravates his condition by refusal of care with intent to reduce his aptitude for service, and augment the chances or the amount of final compensation. In such cases as these any suitable disciplinary measures may be applied. These instructions appear to be based on sound common-sense principles.

### REFUSAL TO SUBMIT TO OPERATIONS.

With regard to more serious operations, it is pointed out in the French Minister of War's official instructions that the right to refuse a bloody operation with or without anæsthesia is considered absolute in law and jurisprudence, because every bloody operation entails a risk of death. The exercise of indisputable right of refusal, however, may entail certain consequences. The wounded may have his final indemnification refused, especially in the following two cases: (*a*) when the operation is very trifling and does not require general anæsthesia; (*b*) when an operation is urgent beyond all doubt to avoid a complication capable of producing an important or absolute incapacity. In such cases the surgeon is advised to give the patient friendly advice that the operation offers the sole chance of survival or of minimum infirmity, and he should also suggest a consultation with one or several surgeons. But if, notwithstanding this, the patient persists in his refusal, the surgeon in charge of the case is instructed to draw up a report giving exact data with reference to the proposed operation, its justification, and the patient's refusal, with his reasons. This report has to be signed by the patient, the surgeon in charge of the case, and the surgeon-in-chief. With this report before them, it is the duty of experts to estimate on a fractional scale the extent to which the operation might have reduced the total incapacity for work. When applications for retirement pension or for relief pension are passed on, the surplus functional incapacity caused by the failure to submit to treatment or operation is to be taken into account.

### THE EASTBY SANATORIUM.

As the result of the refusal of the Bradford Corporation Health Committee to take over the Eastby Sanatorium, some sharp comments were made at a recent meeting of the Bradford Guardians. This sanatorium was the first established by any single Board of Guardians and cost £8,700. The Chairman was of opinion that it was the plain duty of the Corporation Health Committee to have taken over the institution, but now the Guardians felt obliged to accept an offer from Dr. Catherine Arnott, the terms of which were not advantageous to themselves. Dr. Arnott offered to take the sanatorium for three years on a repairing lease. The rent for the first year was to be £100, for the second £150, and £200 for the third year; ten beds for male cases were to be reserved for the Guardians and to be paid for at the rate of £2 per head per week; Dr. Arnott was to have the option of purchase of the building for £4,000. The only other alternative was for the Guardians to continue to use the sanatorium and lose £1,000 a year thereby. Every effort has been made to come to an arrangement with the health committee, and one of the speakers had some severe criticisms to pass on the lack of co-operation between the two departments of the town's administration. It was stated that for early cases of pulmonary tuberculosis there was no provision in Bradford for sanatorium treatment, the cases being dealt with in their own homes. Whatever were the motives actuating the Corporation of Bradford in refusing the Eastby Sanatorium, this course was confirmed after due consideration. The sanatorium has, therefore, been handed over to the charge of Dr. Arnott on the terms above stated.

### BRADFORD AGAIN.

At this same meeting of the Bradford Guardians the Chairman, Mr. R. S. Dawson, pointed out that the tendency of modern legislation and the development of social reform was in the direction of limiting the work of Poor-Law Guardians. The result, he stated, was unnecessary competition between the health committees of Corporations and the Boards of Guardians. Although he agreed that members of the community who required public assistance through no fault of their own should be able to obtain that assistance without the stigma of pauperism, at the same time such public assistance given to excess tended to destroy the independence and self-reliance of the people. Nothing is so likely to produce this result as two authorities competing in relief work, as Bradford demonstrates. The

Guardians, as part of their relief, grant a supply of milk, but the Corporation also give away milk at their depôt, and some families are receiving milk from both sources. Again the Guardians have established at Horton a perfected maternity hospital, and a few yards away the Bradford Corporation has set up a maternity home. Could folly and confusion create worse results than those here recorded at Bradford? Will Mr. Dawson remember that fine phases butter no parsnips, and proceed to action without delay? We always understood that the men of Bradford were canny folk, but fools could do better business than that here recorded.

### HOSPITAL GARDENS.

The garden at the Royal Devon and Exeter Hospital, concerning which we published an interesting article last week, serves as an excellent example of what can be done in this country if proper skill is employed and due care is taken. That gardens can be made to yield a substantial profit was demonstrated, and, what is of at least equal importance, it was shown how an abundance of fresh fruit and vegetables can be supplied to patients; and, as every gardener knows, no vegetables in the world are so good as those he grows himself. But these advantages by no means exhaust all that can be said in favour of hospital gardens. They provide those breathing spaces which are so necessary in convalescence, and they attract the birds whose morning notes are so cheerful to depressed spirits. In natural and healthy surroundings like these the outlook on life is brighter, hopes are raised, and recovery is speedier. There are, of course, some who prefer the ornamental garden, with the marks of the scissors on trees and shrubs; but for the hospital the ideal is the kitchen garden. Besides, in these days, when sometimes a scarcity of food seems to threaten, is it not a patriotic duty to make the soil at our own doors as fruitful as possible? We should be glad to receive from readers of The Hospital photographs and accounts of hospital gardens with which they are familiar.

### SICKNESS BENEFIT IN VOLUNTARY HOSPITALS AND SANATORIA.

Our attention has been drawn to a decision of considerable interest and importance recently given by referees appointed by the Insurance Commissioners in favour of the Reading Insurance Committee. The question in dispute arose through a claim for sickness benefit being made upon an approved society by the Reading Insurance Committee in respect of a woman, without dependants, admitted to Woodhurst Sanatorium, who, until the time of her admission, had remained at work and had not herself claimed the sickness benefit from her society. The society appears to have held that as the woman did not make a claim for the benefit, the Insurance Committee could not do so. The case was, however, decided against the society as already stated. The point is that under the provisions of the Act of 1911 institutional treatment, whether in a hospital or a sanatorium, for persons without dependants, was considered as rendering unnecessary the payment of sickness or disablement benefit to such persons. Hospital treatment was, however, differentiated in the Act from treatment in a sanatorium or other approved institution for the purposes of sanatorium benefit, and in the latter case the sickness or disablement benefit, which would otherwise have been payable to the insured, was made payable to the Insurance Committee for its general purposes. If the insured person had been in hospital and not in a sanatorium she would have been entitled to receive the sickness benefit at the time of her discharge—this privilege being conferred by the Act of 1913. It would in this case have been necessary for the insured to claim the benefit and to produce the necessary medical certificate in support of that claim. It has been suggested that the decision will involve hardship to a large number of insured persons, presumably on the ground that when they are discharged from sanatoria they will find a number of sickness benefit payments recorded against them which they have not received personally. This view of the case, however, appears to overlook the fact that sickness benefit is intended to provide support for the insured person when incapable of work through bodily or mental affliction, and that the insured person receives support while in the sanatorium, which probably costs considerably more than the amount of the sickness benefit. The more logical argument is that the Act of 1913 should be amended to provide that payment of sickness or disablement benefit in respect of insured persons without dependants should be made to the hospitals in which they are inmates instead of being held over to be paid as a lump sum on their discharge. Only by such an amendment can the illogical distinction between voluntary hospitals and sanatoria, involved in Section 12 of the Act of 1911, be remedied.

### THIS WEEK'S DRUG MARKET.

A somewhat quieter tone has been noticeable in the drug market during the past week, but no doubt this is only temporary. So far as synthetic drugs are concerned the falling-off in inquiries is due to extreme scarcity and consequent high prices, and for some time to come no revival of activity in this department is to be expected. Aspirin, salol, benzoic acid, sodium benzoate, phenacetin, and sulphonal are again dearer, while it is becoming increasingly difficult to obtain supplies of resorcin and guaiacol carbonate. The upward tendency in the price of quinine still continues, and the advice given last week, to follow closely the course of this market, may be repeated. The position of opium is practically unchanged. There is no sign of any weakening on the part of holders of cod-liver oil. Hypophosphites are dearer. Oil of sandalwood has an upward tendency in price. Orris root is dearer and is likely to advance still further, owing to the fact that the new crop is short. Makers of bismuth salts, mercurials, iodides, caffeine, morphine, and codeine continue extremely busy.

